# Impaired COMMD10-Mediated Regulation of Ly6C^hi^ Monocyte-Driven Inflammation Disrupts Gut Barrier Function

**DOI:** 10.3389/fimmu.2018.02623

**Published:** 2018-11-14

**Authors:** Odelia Mouhadeb, Shani Ben Shlomo, Keren Cohen, Inbal Farkash, Shlomo Gruber, Nitsan Maharshak, Zamir Halpern, Ezra Burstein, Nathan Gluck, Chen Varol

**Affiliations:** ^1^The Research Center for Digestive Tract and Liver Diseases, Tel-Aviv Sourasky Medical Center and Sackler School of Medicine, Tel-Aviv University, Tel Aviv, Israel; ^2^Department of Clinical Microbiology and Immunology, Sackler School of Medicine, Tel-Aviv University, Tel Aviv, Israel; ^3^Department of Internal Medicine, University of Texas Southwestern Medical Center, Dallas, TX, United States; ^4^Department of Molecular Biology, University of Texas Southwestern Medical Center, Dallas, TX, United States

**Keywords:** COMMD10, Ly6C^hi^ monocytes, inflammasome, systemic inflammation, colitis

## Abstract

Ly6C^hi^ monocyte tissue infiltrates play important roles in mediating local inflammation, bacterial elimination and resolution during sepsis and inflammatory bowel disease (IBD). Yet, the immunoregulatory pathways dictating their activity remain poorly understood. COMMD family proteins are emerging as key regulators of signaling and protein trafficking events during inflammation, but the specific role of COMMD10 in governing Ly6C^hi^ monocyte-driven inflammation is unknown. Here we report that COMMD10 curbs canonical and non-canonical inflammasome activity in Ly6C^hi^ monocytes in a model of LPS-induced systemic inflammation. Accordingly, its deficiency in myeloid cells, but not in tissue resident macrophages, resulted in increased Ly6C^hi^ monocyte liver and colonic infiltrates, elevated systemic cytokine storm, increased activation of caspase-1 and-11 in the liver and colon, and augmented IL-1β production systemically and specifically in LPS-challenged circulating Ly6C^hi^ monocytes. These inflammatory manifestations were accompanied by impaired intestinal barrier function with ensuing bacterial dissemination to the mesenteric lymph nodes and liver leading to increased mortality. The increased inflammasome activity and intestinal barrier leakage were ameliorated by the inducible ablation of COMMD10-deficient Ly6C^hi^ monocytes. In consistence with these results, COMMD10-deficiency in Ly6C^hi^ monocytes, but not in intestinal-resident lamina propria macrophages, led to increased IL-1β production and aggravated colonic inflammation in a model of DSS-induced colitis. Finally, COMMD10 expression was reduced in Ly6C^hi^ monocytes and their corresponding human CD14^hi^ monocytes sorted from mice subjected to DSS-induced colitis or from IBD patients, respectively. Collectively, these results highlight COMMD10 as a negative regulator of Ly6C^hi^ monocyte inflammasome activity during systemic inflammation and IBD.

## Introduction

Macrophages are myeloid immune cells that are strategically positioned throughout the body tissues, where they play key innate immune effector functions during development and adulthood, while orchestrating processes related to tissue maintenance and repair, surveillance of hazardous foreign material and tissue specific functions (1). A long lasting dogma held that tissue-resident macrophages rely on replenishment by bone marrow (BM)-derived monocytes (2). However, it is now well-established that most tissue-resident macrophages are established prenatally and maintained through adulthood by longevity and limited self-renewal (1). Ly6C^hi^ monocytes have instead emerged as an additional highly plastic and dynamic emergency squad that can be rapidly recruited to sites of inflammation and complement classical tissue-resident macrophages by pro-inflammatory or resolving activities (1, 3). In the particular examples of drug-induced liver injury (4, 5) and steatohepatitis (6), recruited inflammatory Ly6C^hi^ monocytes differentiate into an ephemeral macrophage subset that is functionally distinct from the resident Kupffer cell (KC) population. Matching the dynamic gut landscape with its constant tissue renewal, intestinal lamina propria macrophages (lpMF) form a unique paradigm of tissue resident macrophages, as their majority are replenished under homeostatic conditions by circulating Ly6C^hi^ monocytes (7–12). Yet, under inflammatory settings, the same monocytes give rise to distinct effector cells that actively promote gut inflammation (10, 13).

Inflammasomes are key signaling platforms composed of multimeric protein complexes that orchestrate host defense mechanisms in response to pathogenic microorganisms or death-associated molecular patterns (DAMPs). Upon assembly of different inflammasomes, caspase-1 is self-cleaved and subsequently proteolytically activates the pro-inflammatory cytokines IL-1β and IL-18 (14). In general, inflammasome activation employs a two-step mechanism; recognition of a first signal e.g., via Toll-like receptor (TLR) signaling provides transcriptional upregulation of pro-IL-1β, whereas a second signal is recognized for proteolytic activation (15). Recently, a “non-canonical” inflammasome pathway has become evident in which lipopolysaccharide (LPS) from Gram-negative bacteria is directly sensed by cytosolic caspase-11 or its human orthologs caspase-4 and caspase-5 to trigger caspase-1-independent pyroptosis, a form of cell death, and caspase-1-dependent secretion of IL-1β and IL-18 (16, 17). Blood monocytes and macrophages are considered as primary sources of IL-1β. Yet, while macrophages seem to rely on the two-step model, blood monocytes are mechanistically competent of releasing mature IL-1β even following a single stimulus of TLR-2 or−4 (18, 19). Interestingly also, classical human CD14^hi^ monocytes, which are equivalent to the murine Ly6C^hi^ monocytes, produce higher levels of IL-1β in response to LPS in comparison with the non-classical monocyte subset as a result of increased *Il1b* mRNA decay in the latter (20). Therefore, Ly6C^hi^ monocyte dwelling in the circulation and their rapid tissue recruitment in various inflammatory disorders necessitates tight regulation of their inflammasome activation to support appropriate immunity and avoid immune-pathology.

The COMMD (copper metabolism MURR1 domain) protein family includes 10 evolutionarily conserved proteins present in eukaryotic multicellular organisms. All share the structurally conserved COMM domain, which serves as an interface for the regulation of protein-protein interactions. The specific functions of COMMD proteins are poorly defined, but they seem to play distinct and non-redundant roles involved with transcriptional regulation and protein trafficking in various physiological processes (21, 22). COMMD1 is the best characterized member of the family and has been implicated in many different cellular functions, such as copper and cholesterol homeostasis, ionic transport, transcription regulation, and oxidative stress (23). With respect to immune cells, COMMD1-targeted deficiency in myeloid cells results in increased NF-κB activation, thereby exacerbating the course of LPS-induced systemic inflammation and colitis (24). Aside from their effects on transcription factor function, COMMD proteins have been identified as essential components of the COMMD/CCDC22/CCDC93 (CCC) protein complex, which modulates endolysosome architecture and is required for the correct trafficking of different transmembrane proteins that traverse through this compartment (22, 25). Collectively, these features of COMMD proteins mark them as candidate mediators of monocyte and macrophage immune responses. Studies on the specific immunoregulatory role of COMMD10 however, have so far been hindered by the embryonic lethality of COMMD10-knockout mice.

Here we utilized conditional COMMD10-knokcout mice allowing the targeting of COMMD10-deficiency to distinct myeloid immune cells. We show that COMMD10-deficiency dramatically augments canonical and non-canonical inflammasome activation in Ly6C^hi^ monocytes, but not tissue-resident macrophages, thus fueling inflammation in mice with LPS-induced systemic inflammation or colitis.

## Materials and methods

### Mice

Animal experiments were performed with male adult C57BL/6J mice (8–12-weeks old). Animals were maintained in specific pathogen-free animal facility. *LysM*^Δ*Commd*10^ and *Cx3cr1*^Δ*Commd*10^ mice were generated by crossing *Lyz2*^*cre*^ and *Cx3cr1*^*cre*^ (26) mice with Commd10^*fl*/*fl*^ mice, respectively, which were purchased from the EUCOMM consortium (strain EM: 05951) (C57BL/6J background). Experiments with *LysM*^Δ*Commd*10^ and *Cx3cr1*^Δ*Commd*10^ mice were performed on mice heterozygous for these genes.

### Human biopsies and blood monocyte isolation

Intestinal biopsies were obtained from 16 terminal ileums and colons of IBD patients with active disease according to pathological assessment of nearby biopsies and from 14 age and sex-matched healthy controls. Biopsies were immediately frozen in liquid nitrogen for further analyses. Peripheral blood (20 ml) was obtained from 15 IBD patients or healthy controls. PBMCs were enriched from whole blood by Ficoll density gradient media (Ficoll-PaqueTM PLUS, GE Healthcare). CD14^+^CD16^−^HLA-DR^+^ cells were sorted using FACS-Aria machine (BD Biosciences) and used immediately for RNA isolation.

### Ethics statement

Studies with human cells and tissues were carried out in accordance with the recommendations of Tel-Aviv Sourasky Medical Center Helsinki committee. All subjects gave written informed consent in accordance with the Declaration of Helsinki. All procedures involving human subjects were reviewed and approved by the Institutional Review Boards (Tel-Aviv Sourasky Medical Center IRB approval # TLV-0579-10). All mouse studies were carried out in accordance with the recommendations of Tel-Aviv Sourasky Medical Center ethical committee for animal studies. The protocols were approved by the local committee (# 16-6-13).

### BMDM and BM neutrophil preparation and stimulation

BMDM were prepared by flushing BM from the femur and tibia and culturing in RPMI medium containing FBS (10%), penicillin (100 IU/ml), streptomycin (100 μg/ml) and macrophage-colony stimulating factor (M-CSF, 20 ng/ml), at 37°C in 5% CO_2_. Media was supplemented every 2–3 days. On day 6, 500,000 BMDM were seeded and subjected to LPS stimulation (Sigma-Aldrich, L2880 Lot #025M4040V) (100 ng/ml) for 3 h, or to *Escherichia coli* (ATCC 25922) at multiplicity of infection (MOI) = 1, with early log phase bacteria. 30 min after infection cells were washed and supplemented with gentamicin (100 ng/ml) to eliminate extracellular bacteria. At indicated times macrophages were washed twice with PBS^−/−^ (without Ca^++^ and Mg^++^), and collected for western blot analyses. Neutrophils were isolated from BM by magnetic separation using the neutrophil isolation kit (Miltenyi Biotec, Bergisch Gladbach, Germany cat# 130-092-332). Neutrophils were enriched to high purity (above 99%) and identified using flow cytometry as CD11b^+^Ly6G^+^ cells. About 2,000,000 neutrophils were seeded in RPMI containing 10% FBS, 1% L-Glutamine and 0.1% penicillin streptomycin, and incubated for 30 min in a 5% CO_2_ at 37°C. Non-adherent cells were removed and neutrophils were subjected to LPS (100 ng/ml) for 3 h. ATP (InvivoGen, cat# Tlrl-atp1) was added in the last 30 min of the experiment.

### Sorting of splenic Ly6C^hi^ monocytes

Spleens were subjected to mechanical meshing and filtered through 200 μM wire mesh. The cell sediments were washed and pellet was lysed for erythrocytes by 2 min incubation with ACK buffer composed of 0.15 M NH_4_Cl, and 0.01 M KHCO_3_, and then washed again with PBS^−/−^ (without Ca^2+^ and Mg^2+^). Ly6C^hi^ monocytes were defined as CD45^+^CD11b^+^CD115^+^ Ly6C^hi^ and sorted to high purity (above 90%) using the SH800 sorter machine (Sony Biotechnology Inc). Cells were subjected to LPS as detailed above for BMDM.

### Lymphocyte isolation from spleen and thymus

Spleen and thymus were subjected to mechanical meshing into a sterile RPMI containing 10% FBS, 1% L-Glutamine and 0.1% penicillin streptomycin. Erythrocytes were lysed by incubation with ACK buffer for 2 min. Cells were plated and selective adherence to plastic was used to enrich non-adherent lymphocytes.

### LPS-induced systemic inflammation

Mice at the same range of body mass were i.p. injected with LPS, 0.2 mg/mouse, in 100 μl saline. Mice were sacrificed at indicated time points.

### DSS-induced colitis

Dextran sulfate sodium (DSS, MP Biomedicals, cat# 160110) was administered in drinking water (1.5%) for 4 and 7 days. Colitis severity was assessed using the murine colonoscopy system (IMAGE1 SPIES™ series, Karl Storz, Germany). Quantification of colitis severity was graded as previously described (27). Digitally recorded video files were processed with Windows Movie Maker software (Microsoft).

### Inducible ablation of Ly6C^hi^ monocytes

In the LPS-induced systemic inflammation experiments, some of the mice received an i.p. injection of 400 μl anti-mouse CCR2 mAb (clone MC-21)-conditioned media (30 μg Ab/ml), as previously described. Injections were started at 12 h prior to LPS challenge, and repeated every 24 h until sacrifice. In the DSS-induced colitis experiment, MC-21 was injected every 24 h starting at day 1 of DSS, as previously described (10).

### Isolation of hepatic non-parenchymal cells

Hepatic non-parenchymal cells were isolated from liver as previously described (4). In brief, mice were perfused with 10 ml of cold PBS^−/−^ and the liver was excised. Livers were cut into small fragments, incubated with shaking (37°C, 250 rpm for 45 min) with 5 ml digestion buffer [5% FCS, 0.5 mg/ml collagenase VIII (Sigma-Aldrich, Rehovot, Israel, C5138-500MG) in PBS^+/+^ (with Ca^2+^ and Mg^2+^)]. Subsequently, livers were subjected to mechanical meshing and filtered through 200 μM wire mesh. This was followed by three cycles of washings with PBS^−/−^ at 40 g from which the supernatant was taken, omitting the parenchymal cell pellet. The supernatant was then centrifuge (400 g) and cell pellet was lysed for erythrocytes by 2 min incubation with ACK buffer (0.15 M NH_4_C and 0.01 M KHCO_3_).

### Isolation of colonic lamina propria cells

Colonic lamina propria phagocytes were isolated as previously described (10). In brief, extra-intestinal fat tissue was carefully removed and colons were then flushed of their luminal content with cold PBS^−/−^, opened longitudinally, and cut into 3–5 mm pieces. Epithelial cells and mucus were removed by 40 min incubation with 5 ml HBSS^−/−^ (without Ca^2+^ and Mg^2+^) containing 5% FBS, 2 mM EDTA, and 1 mM DTT (Sigma) at 37°C shaking at 250 rpm. Colon pieces were then incubated with shaking (37°C, 250 rpm for 45 min) with 5 ml digestion buffer [5% FCS, 1 mg/ml collagenase VIII (Sigma-Aldrich, Rehovot, Israel, C5138-500MG) in PBS^+/+^]. The cell suspension was then filtered through 200 μM wire mesh and washed with PBS^−/−^.

### Protein immunoblotting

Total protein from BMDM, neutrophils, lymphocytes and mentioned tissues was extracted in ice cold RIPA buffer (C-9806S, Cell Signaling Tech. Beverly, Massachusetts) containing protease inhibitors (P8340, Sigma Aldrich St. Louis, Missouri). Proteins were detected by immunoblotting using standard techniques. Antibodies that were used: caspase-11 (sc-374615), GAPDH (sc-47724), β-ACTIN (sc-47778), Pol II (sc-9001), GCN5 (sc-20698) from Santa Cruz; caspase-1(AG-20B-0042) from adipogen; anti-COMMD10 antibody (GTX121488) from Genetex, pP65 (3033) and P65 (6956) from Cell Signaling. Blots were incubated with HRP-conjugated secondary antibodies, and subjected to chemiluminescent detection using the MicroChemi imaging system (DNR Bio-Imaging Systems, Israel). P65 subcellular fractionation was performed using NE-PER nuclear/cytoplasmic extraction kit (78835, Thermo scientific, Paisley, UK) per manufacturer's instructions. Equivalent protein amounts were loaded for both nuclear and cytoplasmic fractions.

### *Commd10* gene silencing in human monocyte derived macrophages

Human blood CD14^+^ monocytes were isolated using the magnetic monocyte isolation kit II (Miltenyi Biotec, Bergisch Gladbach, Germany cat# 130-091-153). Cells were cultured in RPMI medium containing FBS (10%), penicillin (100 IU/ml), streptomycin (100 μg/ml) and macrophage-colony stimulating factor (M-CSF, 20 ng/ml), at 37°C in 5% CO_2_. Media was supplemented every 2–3 days. On day 6 cells were plated in 6-well plates (~5 × 10^5^ cells/well) for 18–24 h before transfection. Cells were transfected with 200 nM ON-TARGETplus SMARTpool siRNA targeting *Commd10* or with non-targeting scrambled siRNA [Thermo Scientific, Dharmacon (Illkirch, France)] using HiPerFect transfectant reagent (Qiagen, Courtaboeuf, France) for 48 h, per manufacturer's instructions. Cells were then stimulated with 100 ng/ml LPS for 1 h.

### Quantitative real-time PCR (RT-PCR)

Total RNA was extracted from tissues with TRIzol® reagent (Invitrogen, United States), and from BMDM or sorted Ly6C^hi^ monocytes using the RNeasy Mini Kit (QIAGEN, Germany). RNA was reverse transcribed with a high-capacity cDNA reverse transcription kit (Applied Biosystems, Foster City, California). All PCR reactions were performed with SYBR Green PCR Master Mix kit (Applied Biosystems) and Applied Biosystems 7300 Real-Time PCR machine. Quantification of PCR signals of each sample was performed by the ΔCt method normalized to *Gapdh* housekeeping gene. For the list of gene specific primers see Table S1.

### Flow cytometry analysis

The following anti-mouse antibodies were used (dilutions are indicated): CD45 (clone 30-F11, 1:100), CD11b (clone M1/70, 1:300), Ly6C (clone HK1.4, 1:300), CD115 (clone AFS98, 1:100), CD64 (clone X54-5/7.1, 1:50), Ly6G (clone 1A8, 1:100), and Tim4 (clone RMT4-54, 1:50), MHCII (clone M4-114.15.2, 1:200), CX_3_CR1 (clone SA011F11, 1:100), CD14 (clone M5E2, 1:100), CD16 (clone 3G8, 1:100) all purchased from BioLegend, San Diego, CA, USA. Anti-mouse F4/80 (clone A3-1, 1:50) was purchased from BIORAD. Cells were analyzed with BD FACSCanto™ II (BD Bioscience). Flow cytometry analysis was performed using FlowJo software (TreeStar, Ashland, OR, United States).

### ELISA

Mouse plasma and supernatants from BMDM, BM neutrophils or sorted Ly6C^hi^ monocytes were collected and kept in −80°C until assessed. The levels of the cytokines TNFα, IL-1β, and IFNγ were assessed with the DuoSet ELISA kit (R&D Systems). Plasma from mice at 4 h following LPS challenge was subjected to multiplex cytokine array using the mouse cytokine array Q1 (R&D Systems), and analyzed by Q-analyzer software for QAM-CYT-1.

### Fitc dextran assay for intestinal barrier function

Mice received FITC-conjugated dextran by gavage (Sigma-Aldrich, Cat# FD4, 160 mg/100 g of body mass) 20 h after LPS stimuli. Four hours later, plasma was collected and FITC fluorescence signal was analyzed using the Synergy 2 Multi- Mode Reader (BioTek Instruments Inc).

### Colony formation unit (CFU) assay

Tissues were weighed and briefly homogenized (Polytron PT-MR 2100, Kinematica AG) in 1 ml PBS^−/−^. Appropriate dilutions were seeded on LB agar plates and incubated at 37°C for 24 h. The number of colonies was counted and CFU per gram-tissue mass was calculated.

### Data analysis using the immgen database

Raw microarray data were downloaded from the Immunological Genome Project data Phase 2 (GSE37448 microarray datasets) using Partek GS 6.6 (http://www.partek.com/pgs). Blood Ly6C^hi^ monocytes (*n* = 3) and neutrophils (*n* = 4) were selected. Raw gene expression data were obtained for *Commd10*. The gene expressions of the adipocyte marker adiponectin (*Adipoq*) and of the hepatocyte and cholangiocyte marker Cytokeratin18 (*Krt18*) were also obtained to set background expression levels.

### Statistical analysis

In most experiments, statistical differences between two groups were determined using the unpaired two tailed *t*-test with GraphPad. Survival data (**Figure 2C**) were analyzed by Logrank (Mental-cox method) and Gehan-Breslow-Wilcoxon tests using GraphPad, comparing the *Commd10*^*fl*/*fl*^ and *LysM*^Δ*Commd10*^ groups. Significance was defined if *p*-value was <0.05 as following: ^*^p < 0.05; ^**^p < 0.01; ^***^p < 0.001.

## Results

### COMMD10-deficient monocyte-derived macrophages exhibit increased pro-inflammatory response to LPS

**Figure 1:**
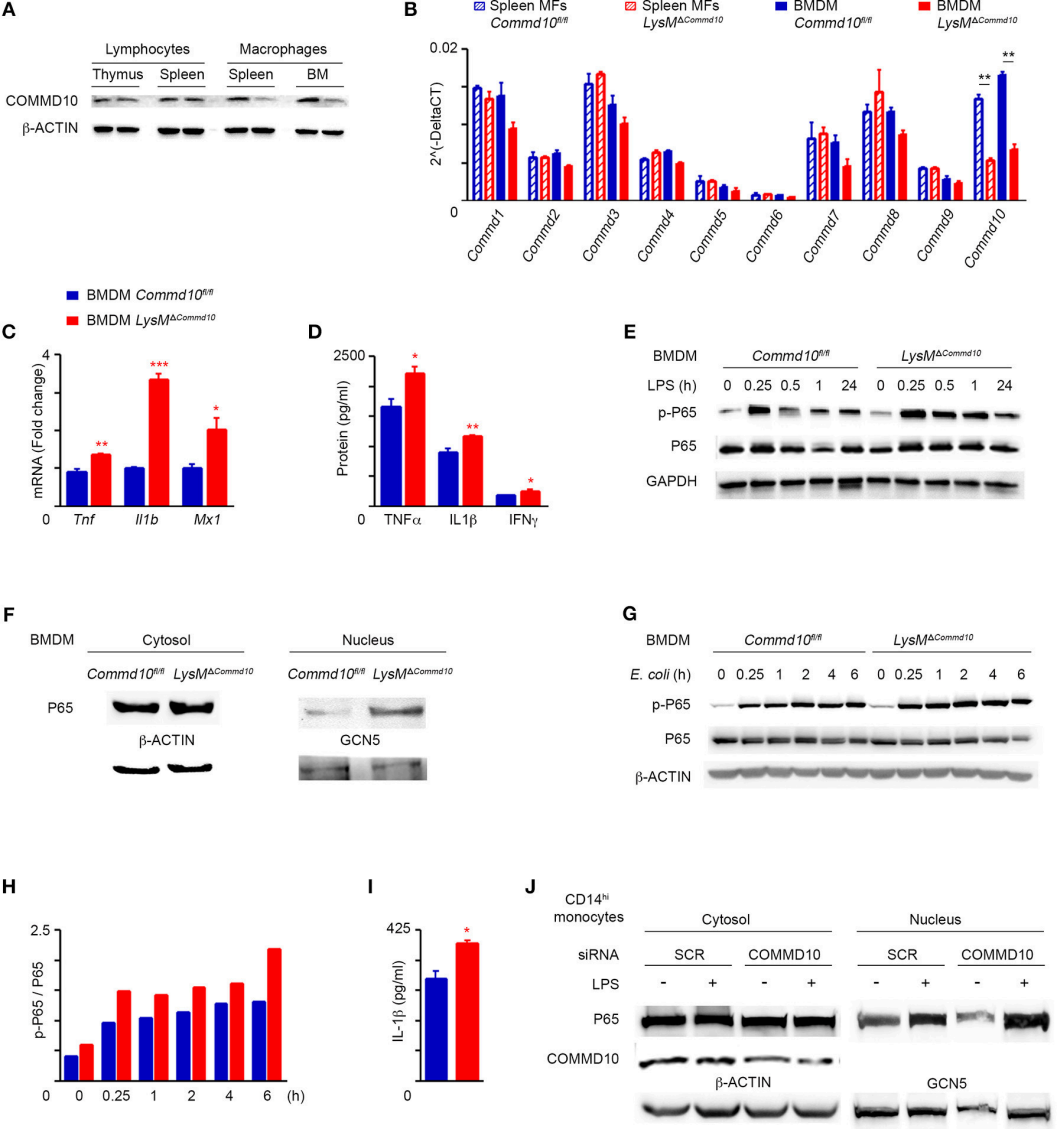
*LysM*^Δ*Commd*10^ BMDM respond to LPS by higher production of pro-inflammatory cytokines. **(A,B)** Cells were isolated from spleen and BM of *Commd10*^*fl*/*fl*^ (blue) or *LysM*^Δ*Commd*10^ (red) mice. **(A)** Immunoblots showing the expression of COMMD10 in cell lysates of thymic or splenic lymphocytes vs. splenic or BM-derived macrophages. β-actin was utilized as control. **(B)** qRT-PCR gene expression of all COMMD family members in splenic macrophages and BMDM (*n* = 3). **(C,D)** BMDM were stimulated with LPS (100 ng/ml) for 3 h. **(C)** qRT-PCR gene expression of pro-inflammatory cytokine in cell lysate (*n* = 3). **(D)** ELISA of pro-inflammatory cytokine levels in cell free supernatants (*n* = 3). **(E)** Representative immunoblot of BMDM cell lysates following LPS challenge (100 ng/ml) showing the protein expression of phospho-P65 (p-P65) and P65 over time course. GAPDH was used as control. **(F)** Immunoblots showing P65 expression in cytosolic and nuclear fraction lysates of LPS-treated BMDM. β-ACTIN and Polymerase II antibodies, respectively, were used as controls (*n* = 3). **(G–I)** BMDM from *Commd10*^*fl*/*fl*^ or *LysM*^Δ*Commd*10^ mice were infected with *E. Coli* at multiplicity of infection (MOI) = 1. **(G)** Immunoblots showing the protein expression of p-P65 and P65 over time course. β-ACTIN was used as control. **(H)** Respective densitometry-based quantification of p-P65/P65 ratio in *Commd10*^*fl*/*fl*^ vs. *LysM*^Δ*Commd*10^ BMDM over time course following *E. coli* infection. **(I)** ELISA analysis of IL-1β from supernatants at 6 h post-infection with *E. coli* (*n* = 4). **(J)** Immunoblots showing cytosolic COMMD10 protein expression following siRNA-based gene silencing vs. scrambled siRNA control, β-ACTIN was used as control, and immunoblots showing P65 expression in cytosolic and nuclear fraction lysates of LPS-treated human CD14^hi^ monocyte-derived macrophages. GCN5 was used as control in the nuclear fraction (*n* = 3). Data were analyzed by unpaired, two-tailed *t-*test, comparing each time between *Commd10*^*fl*/*fl*^ and *LysM*^Δ*Commd*10^ (red stars), and are presented as mean ± SEM with significance: ^*^p < 0.05, ^**^p < 0.01, ^***^p < 0.001. Data in **(A,B,E)** represents two-independent experiments. Data in **(C,D,F–J)** are from a single experiment.

The role of COMMD proteins in the regulation of myeloid cell function has been so far overlooked, in particular with respect to COMMD10, given the embryonic lethality of COMMD10-knockout mice. Therefore, we generated conditional COMMD10 knockout mice (*Commd10*^*fl*/*fl*^) that allow specific targeting of COMMD10-deficiency to myeloid cells by crossing them with *Lyz2*^*cre*^ mice. The resulting *LysM*^Δ*Commd10*^ mice exhibited normal birth rate and life expectancy (*data not shown*). Efficient COMMD10 protein-deficiency was evident in macrophages generated from the spleen and BM, but not in lymphoid cells extracted from the spleen and thymus (Figure 1A). Both splenic and bone marrow-derived macrophages (BMDM) exhibited specific reduction in *Commd10* expression, while the expression of all other *Commd* genes was intact (Figure 1B). Therefore, the *LysM*^Δ*Commd10*^ mice provided us with a model to study the immunoregulatory role of COMMD10 in myeloid cells. We assessed the effect of COMMD10-deficiency on LPS-induced innate immune responses in BMDM. LPS-stimulated BMDM from *LysM*^Δ*Commd10*^ mice showed increased transcription of pro-inflammatory cytokines including TNFα (*Tnf)*, IL-1β (*Il1b*) and the MX Dynamin Like GTPase 1 (*Mx1*) (Figure 1C). Supernatants from overnight cultures of *LysM*^Δ*Commd10*^ BMDM contained higher levels of the secreted cytokines TNFα, IFNγ and IL-1β (Figure 1D). Therefore, COMMD10-deficiecny in Ly6C^hi^ monocyte-derived BMDM exhibit increased inflammatory cytokine production in response to LPS.

COMMD1, the prototype member of the COMMD family, was initially reported to inhibit NF-κB-mediated transcription (28). Further studies in human embryonic kidney cells genetically manipulated to overexpress distinct COMMD proteins revealed that COMMD10 is also capable of inhibiting TNF-mediated NF-κB activation (21). Yet, the physiological relevance of this finding to immune cells, specifically monocytes, remains elusive. In this respect, LPS-challenged COMMD10-deficient BMDM exhibited intensified and more persistent phosphorylation of P65 (Figure 1E) as well as increased translocation of P65 to the nucleus (Figure 1F). Increased levels of phosphorylated P65 (pP65) and IL-1β secretion were also evident in COMMD10-deficient BMDM infected with the Gram^neg^ bacteria *Escherichia coli* (*E. coli*) (Figures 1G,H,I). To further validate these findings in human CD14^hi^ monocytes, which are the equivalent of mouse Ly6C^hi^ monocytes, we utilized siRNA to silence *Commd10* gene expression in M-CSF-differentiated CD14^hi^ monocyte-derived macrophages. Even partial reduction in COMMD10 expression was sufficient to induce greater activation of NF-κB in response to LPS, as manifested by increased nuclear translocation of P65 (Figure 1J). Therefore, these results establish a role for COMMD10 in suppressing NF-κB activation.

### COMMD10-deficiency in myeloid cells exacerbates LPS-induced systemic inflammation

**Figure 2:**
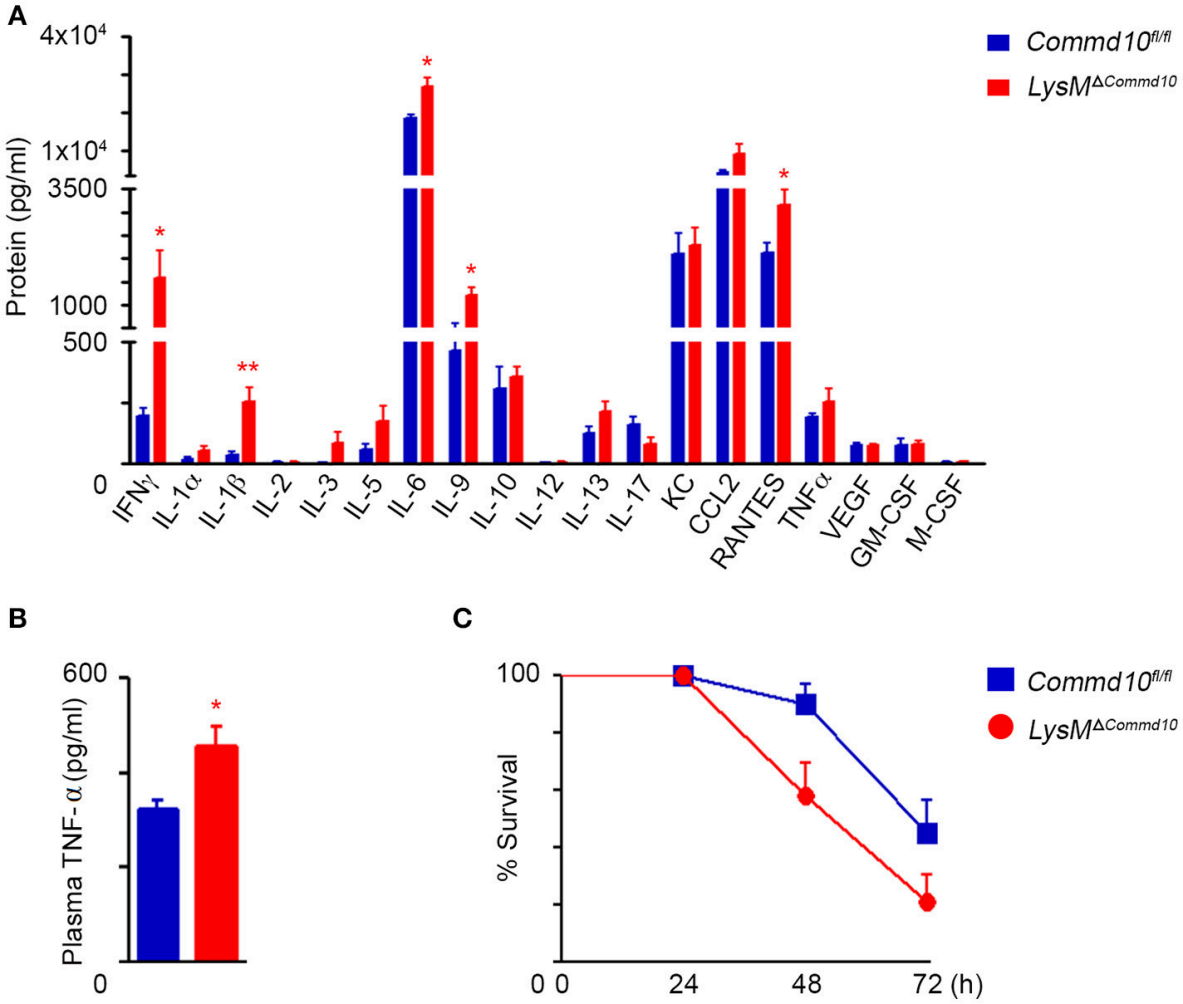
*LysM*^Δ*Commd*10^ mice exhibit aggravated cytokine storm and mortality in response to systemic LPS challenge. **(A,B)** *Commd10*^*fl*/*fl*^ (blue) and *LysM*^Δ*Commd*10^ (red) mice were i.p. injected with LPS (0.2 mg per mouse matched for body mass) and sacrificed 4 h later. **(A)** Multiplex ELISA array of plasma pro-inflammatory cytokines (*n* = 4). **(B)** ELISA analysis of plasma TNF-α (*n* ≥ 3). **(C)** Survival curve over 72 h (*n* = 10). Data in **(A,B)** were analyzed by unpaired, two-tailed *t-*test, comparing each time between *Commd10*^*fl*/*fl*^ and *LysM*^Δ*Commd*10^ (red stars), and are presented as mean ± SEM with significance: ^*^p < 0.05, ^**^p < 0.01. Data in **(C)** were analyzed by Logrank (Mental-cox method) (*P* = 0.03) and Gehan-Breslow-Wilcoxon (*P* = 0.02) tests comparing between the *Commd10*^*fl*/*fl*^ and *LysM*^Δ*Commd10*^ groups. Data in **(A,B)** represent a single experiment. Data in **(C)** represent three independent experiments.

We next examined the response of *LysM*^Δ*Commd10*^ mice to systemic LPS-induced systemic inflammation. Analysis of an array of plasma cytokines at 4 h following LPS challenge revealed higher levels of pro-inflammatory cytokines and chemokines including IFNγ, IL-6, IL-9, CCL5 (RANTES) and IL-1β in the *LysM*^Δ*Commd10*^ vs. *Commd10*^*fl*/*fl*^ mice (Figure 2A). More direct analysis of TNFα levels further revealed its elevated expression in the plasma of *LysM*^Δ*Commd10*^ mice (Figure 2B). This augmented cytokine storm witnessed in the *LysM*^Δ*Commd10*^ mice may induce tissue damage and impair survival. Indeed, mortality rates of *LysM*^Δ*Commd10*^ mice were significantly higher over a period of 72 h in comparison with *Commd10*^*fl*/*fl*^ control mice (*p* = 0.02; Figure 2C). These results suggest that COMMD10-deficiency induces a hyper-inflammatory state that impairs overall survival following acute endotoxic shock.

### COMMD10 negatively regulates inflammasome activity in Ly6C^hi^ monocytes during LPS-induced systemic inflammation

**Figure 3:**
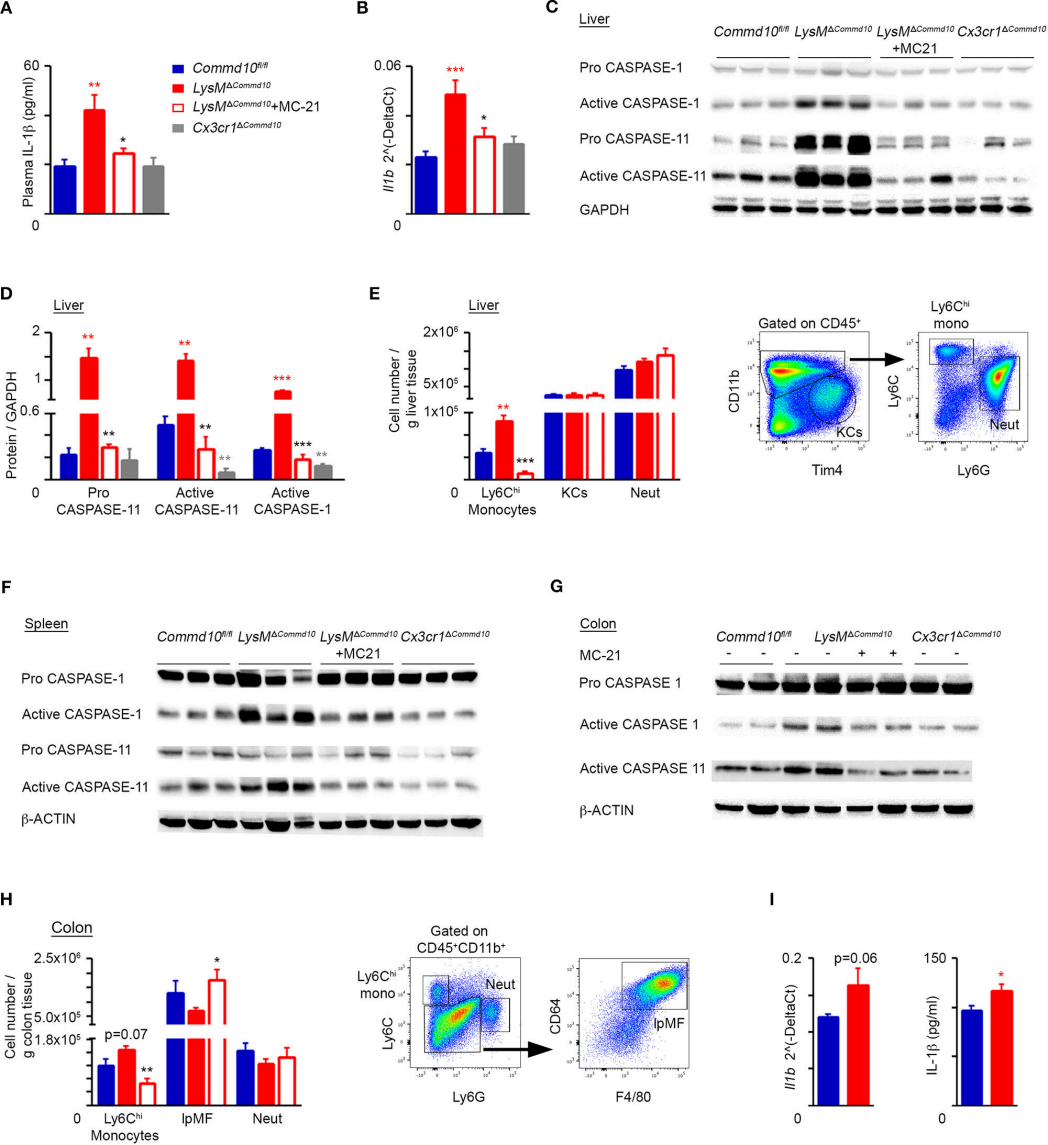
Increased canonical and non-canonical inflammasome activity in COMMD10-deficient Ly6C^hi^ monocytes during LPS-induced systemic inflammation. **(A–H)** *Commd10*^*fl*/*fl*^ (blue), *LysM*^Δ*Commd*10^ (red) or *Cx3cr1*^Δ*Commd*10^ (gray) mice were i.p. injected with LPS (0.2 mg per mouse of similar body mass) and sacrificed 4 h later. Where indicated, MC-21 was injected 12 h prior to LPS stimulation (red border). **(A)** ELISA analysis of plasma IL-1β (*n* ≥ 5). **(B)** qRT-PCR gene expression of *Il1b* in liver extracts (*n* ≥ 5). **(C)** Immunoblots of liver lysates demonstrating the expression of pro- and active- caspase-1 and−11. GAPDH was utilized as control (*n* = 3). **(D)** Respective densitometry-based quantification of pro-caspase-11, active caspase-1 and active caspase-11, normalized to GAPDH. **(E)** Left panel: flow cytometry-based assessment of liver Ly6C^hi^ monocyte, KC and neutrophil abundance normalized to tissue mass (g) (*n* = 5). Right panel: representative flow cytometry images showing gating strategy of liver Ly6C^hi^ monocytes, KCs and neutrophils (*n* = 5). **(F,G)** Immunoblots of **(F)** spleen and **(G)** colon lysates demonstrating the expression of pro- and/or active- caspase-1 and−11. β-actin was utilized as control (*n* = 3 for **E**, *n* = 2 for **F**). **(H)** Left panel: flow cytometry-based assessment of colonic Ly6C^hi^ monocyte, resident lpMF and neutrophil abundance normalized to tissue mass (g) (*n* = 5). Right panel: representative flow cytometry images showing gating strategy of colonic Ly6C^hi^ monocytes, lpMFs and neutrophils out of CD45^+^CD11b^+^ cells (*n* = 5). **(I)** Circulating Ly6C^hi^ monocytes were sorted from their splenic reservoir and stimulated with LPS (100 ng/ml) for 10 h. Left panel: qRT-PCR gene expression of *Il1b*. Right panel: ELISA analysis of IL-1β from cell free supernatants (biological repeats: *n* = 3 for *Commd10*^*fl*/*fl*^ and *n* = 4 for *LysM*^Δ*Commd10*^, each repeat is composed from a pool of three mice). Data were analyzed by unpaired, two-tailed *t-test*, comparing each time between one of the mouse groups with *Commd10*^*fl*/*fl*^ mice (colored stars), or between *LysM*^Δ*Commd*10^ mice with and without MC-21 treatment (black stars). Results are presented as mean ± SEM with significance: ^*^p < 0.05, ^**^p < 0.01, ^***^p < 0.001. Data in **(A–C,E,F,H)** represent two independent experiments. Data in **(D,G)** represent a single experiment.

Inflammasome activation in macrophages is essential to ensure adequate host defense against invading microbes, yet it must be finely controlled to avoid overt tissue damage (14). While both blood monocytes and macrophages are prime producers of IL-1β, monocytes naturally express higher levels of activated caspase-1 allowing its maturation and release even following a single stimulus of TLR-2 or−4 (18). To determine whether Ly6C^hi^ monocytes were responsible for the excessive IL-1β production in the serum of LPS-challenged *LysM*^Δ*Commd10*^ mice (Figure 2A), we induced their ablation by preemptive treatment with the anti-CCR2 MC-21 antibody (10, 29). Indeed, MC-21 treatment specifically depleted Ly6C^hi^ monocytes tissue infiltrates in the liver (Figure S1A) and colon (Figure S1B) of LPS-challenged mice, but was inert to tissue-resident macrophages or neutrophils in these compartments. In addition, we crossed *Commd10*^*fl*/*fl*^ with *Cx3cr1*^*cre*^ mice giving rise to *Cx3cr1*^Δ*Commd10*^ mice. In these mice, cre-driven recombination (and the ensuing COMMD10-deficiency) is efficient in tissue-resident macrophages, significantly less in circulating CX_3_CR1^lo^Ly6C^hi^ monocytes and hardly affects CX_3_CR1^neg^ neutrophils (26). Plasma IL-1β levels were profoundly elevated in *LysM*^Δ*Commd10*^ vs. *Commd10*^*fl*/*fl*^ mice at 4 h following LPS challenge (Figure 3A). Strikingly, this elevation was ameliorated following Ly6C^hi^ monocyte ablation (Figure 3A). Moreover, plasma IL-1β levels were similar in both *Commd10*^*fl*/*fl*^ and *Cx3cr1*^Δ*Commd10*^ mice (Figure 3A). These phenomena were mirrored in the transcription of *Il1b* in the liver (Figure 3B). The activation of caspases-1 and-11 can both drive IL-1β release (15, 16). In this respect, in comparison with *Commd10*^*fl*/*fl*^ mice, there was significantly higher expression of pro-caspase-11 as well as activation of both caspase-1 and-11 in the liver of *LysM*^Δ*Commd10*^, but not *Cx3cr1*^Δ*Commd10*^ mice, at 4 h following LPS challenge (Figures 3C,D). Strikingly, MC-21-mediated Ly6C^hi^ monocyte ablation completely abolished the activation of these caspases in the liver (Figures 3C,D). In alignment, flow cytometry analysis showed increased infiltration of Ly6C^hi^ monocytes, defined as CD11b^+^Ly6C^hi^Ly6G^−^, to the livers of *LysM*^Δ*Commd10*^ vs. *Commd10*^*fl*/*fl*^ mice. In contrast, COMMD10-deficiency had no effect on the abundancy of resident CD11b^int^F4/80^+^Tim4^+^ KCs or infiltrating CD11b^+^Ly6G^+^Ly6C^lo^ neutrophils (Figure 3E). Similarly, increased activation of caspases-1 and-11 was observed in *LysM*^Δ*Commd10*^ spleen (Figure 3F) and colon (Figure 3G), and in both cases, it was ameliorated by MC-21-driven ablation of Ly6C^hi^ monocytes. Moreover, there was a trend for increased infiltration of Ly6C^hi^ monocytes to the *LysM*^Δ*Commd10*^ colon with no clear difference in the abundance of CD11b^+^F4/80^+^CD64^+^ resident lpMF and Ly6G^+^ neutrophils (Figure 3H). To directly delineate the effect of COMMD10-deficiency on inflammasome activation in mature circulating Ly6C^hi^ monocytes, these cells were sorted to high purity from their splenic reservoir according to their simultaneous expression of CD45, CD11b, CD115 (Csf-1R) and Ly6C (*data not shown*). In the absence of COMMD10, Ly6C^hi^ monocytes exhibited a strong trend toward increased transcription of *Il1b* and significantly increased secretion of IL-1β in response to LPS (Figure 3I). Of note, in contrast to Ly6C^hi^ monocytes, there were reduced levels of IL-1β production in the supernatants of LPS and adenosine triphosphate (ATP)-stimulated COMMD10-deficient BM neutrophils (Figures S2A,B). Using published data sets available from the ImmGen Consortium database we could further show that *Commd10* gene expression was near background levels in blood neutrophils and significantly higher in blood Ly6C^hi^ monocytes (Figure S3). Taken together, these findings implicate COMMD10 as a negative regulator of inflammasome activity in Ly6C^hi^ monocytes, but not in tissue-resident macrophages and neutrophils, during LPS-induced systemic inflammation.

### COMMD10-deficiency in Ly6C^hi^ monocytes leads to increased intestinal barrier dysfunction in LPS-induced systemic inflammation

**Figure 4:**
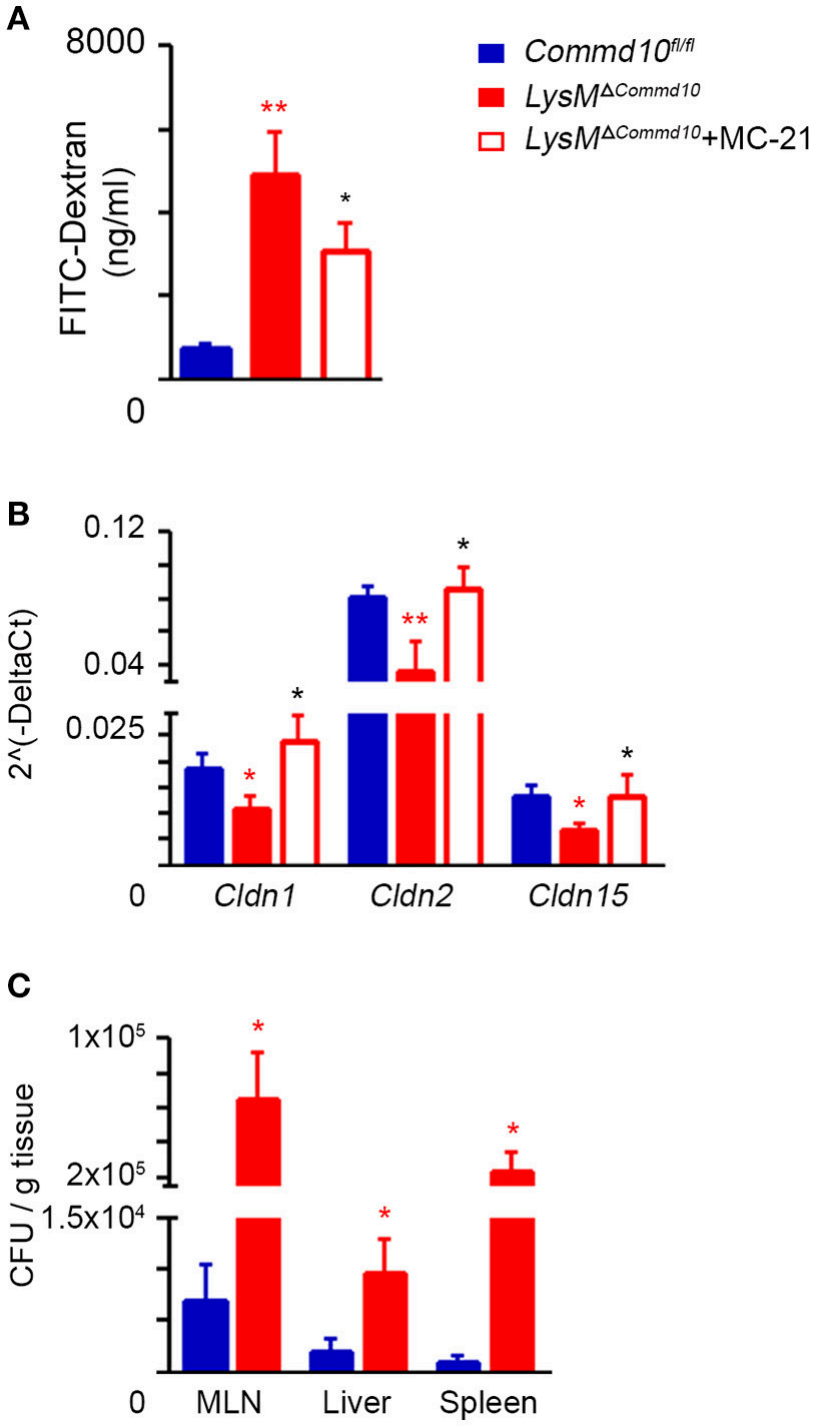
Increased intestinal barrier dysfunction in LPS-challenged *LysM*^Δ*Commd*10^ mice driven by Ly6C^hi^ monocytes. *Commd10*^*fl*/*fl*^ (blue) and *LysM*^Δ*Commd*10^ (red) mice were i.p. injected with LPS (0.2 mg per mouse of similar weight) and sacrificed 24 or 48 h later. Where indicated, MC-21 was injected 12 h prior to LPS stimulation and every 24 h (red border). **(A)** Fluorometric assessment of FITC dextran plasma signal at 4 h following its oral administration to mice after 24 h of LPS challenge (*n* ≥ 6). **(B)** Graphical summary of qRT-PCR assessment of *Claudin 1, 2*, and *15* gene expressions at 48 h following LPS challenge (*n* ≥ 7). **(C)** Indicated tissues were extracted at 48 h following LPS injection. Colony forming units (CFU) were determined and normalized to tissue mass (*n* = 4). Data were analyzed by unpaired, two-tailed *t-*test, comparing each time between *Commd10*^*fl*/*fl*^ and *LysM*^Δ*Commd*10^ (red stars) or between *LysM*^Δ*Commd*10^ mice with and without MC-21 treatment (black stars). Data are presented as mean ± SEM with significance: ^*^p < 0.05, ^**^p < 0.01. Data in **(A–C)** represent 2–3 independent experiments.

Defective intestinal epithelial tight junction (TJ) barrier has been implicated as an important pathogenic factor in sepsis (30). It may lead to bacterial translocation from the intestinal lumen to the mesenteric nodes, liver and systemic circulation, and induce severe complications resulting in high mortality rates. Excess production of inflammatory cytokines, and specifically IL-1β, can damage TJ protein expression and function (31, 32). Therefore, we hypothesized that the increased mortality rates of LPS-challenged *LysM*^Δ*Commd10*^ mice (Figure 2C) may be the result of impaired intestinal barrier function. Indeed, a significant breach in barrier function was evident in *LysM*^Δ*Commd10*^ mice at 24 h following LPS challenge, as confirmed by markedly increased intestinal translocation of the orally administered fluorescent probe FITC-dextran (Figure 4A). Ly6C^hi^ monocyte depletion partially mitigated barrier leakage (Figure 4A). In agreement with the impaired barrier function, expression of colonic tight-junction claudin genes *Cldn* 1, 2 and 15 was decreased in the colons of *LysM*^Δ*Commd10*^ mice at 48 h following LPS challenge (Figure 4B). Ly6C^hi^ monocyte depletion restored their expression to the level present in *Commd10*^*fl*/*fl*^ colons (Figure 4B). In alignment with their increased mortality (Figure 2A), bacterial counts were elevated in mesenteric lymph nodes (MLN), liver and spleen of *LysM*^Δ*Commd10*^ mice (Figure 4C). Collectively, these findings mark an important role for COMMD10 in the protection of intestinal barrier function from Ly6C^hi^ monocyte-driven inflammation during sepsis.

### COMMD10 restrains inflammasome activity in Ly6C^hi^ monocytes during colitis

**Figure 5:**
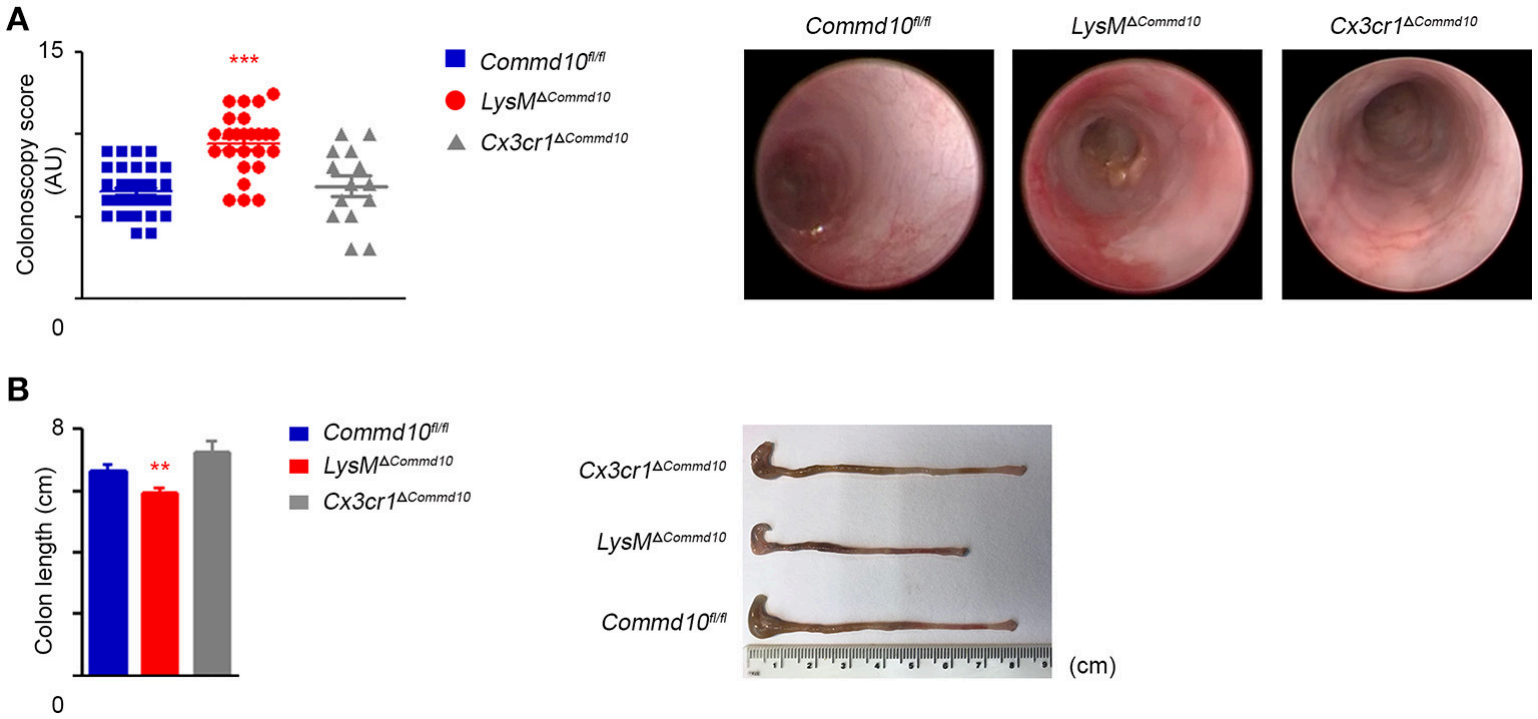
*LysM*^Δ*Commd*10^, but not *Cx3cr1*^Δ*Commd10*^ mice exhibit augmented DSS-induced colitis. *Commd10*^*fl*/*fl*^ (blue), *LysM*^Δ*Commd*10^ (red) or *Cx3cr1*^Δ*Commd*10^ (gray) mice were treated with DSS (1.5% in drinking water) for 7 days. **(A)** Left panel: Scatter plot graph demonstrating colonoscopy scores as determined by live colonoscopy (*n* ≥ 14). Right panel: Representative colonoscopy images. **(B)** Left panel: graph demonstrating colon length. Right panel: Representative colon images (*n* ≥ 17 for *Commd10*^*fl*/*fl*^ and *LysM*^Δ*Commd*10^ groups, *n* = 5 for *Cx3cr1*^Δ*Commd*10^ group). Data were analyzed by unpaired, two-tailed *t*-test, comparing each time between one of the mouse groups with *Commd10*^*fl*/*fl*^ mice (colored stars). Results are presented as mean ± SEM with significance: ^**^p < 0.01, ^***^p < 0.001. Data in **(A,B)** represent 2–3 independent experiments.

The majority of colonic-resident lpMFs rely on constant replenishment by blood Ly6C^hi^ monocytes attracted by local tonic low-grade inflammatory stimuli (8, 9, 11, 12). Newly arriving Ly6C^hi^ blood monocytes are normally conditioned by IL-10 to acquire a non-inflammatory gene-expression profile, while differentiating into CX_3_CR1^hi^ lpMFs (33, 34). However, this conditioning process fails when they enter the inflamed gut, and hence differentiate instead into pro-inflammatory CX_3_CR1^lo^ effector cells that actively promote gut inflammation (10, 13). Interestingly, IL-1β was recently shown to be the key mediator driving intestinal inflammation in mice and patients with IL-10 receptor deficiency (35). Therefore, we next studied the effect of COMMD10-deficiency in myeloid cells on intestinal inflammation in an acute model of dextran sodium sulfate (DSS)-induced colitis. Remarkably, live colonoscopy assessment revealed significantly aggravated colitis in *LysM*^Δ*Commd10*^ vs. *Commd10*^*fl*/*fl*^ control mice (Figure 5A; Movie S1). In the colon of *Cx3cr1*^*cre*^ mice, cre-mediated recombination is mainly targeted to resident lpMFs (34). Notably, the colitis score of *Cx3cr1*^Δ*Commd10*^ mice was similar to that of *Commd10*^*fl*/*fl*^ control mice (Figure 5A; Movie S1), suggesting that COMMD10 does not play a critical anti-inflammatory role in CX_3_CR1^hi^ lpMFs. The aggravated colitis in *LysM*^Δ*Commd10*^ mice was also reflected in the reduced length of their colon in comparison with that of *Commd10*^*fl*/*fl*^ or *Cx3cr1*^Δ*Commd10*^ (Figure 5B).

**Figure 6:**
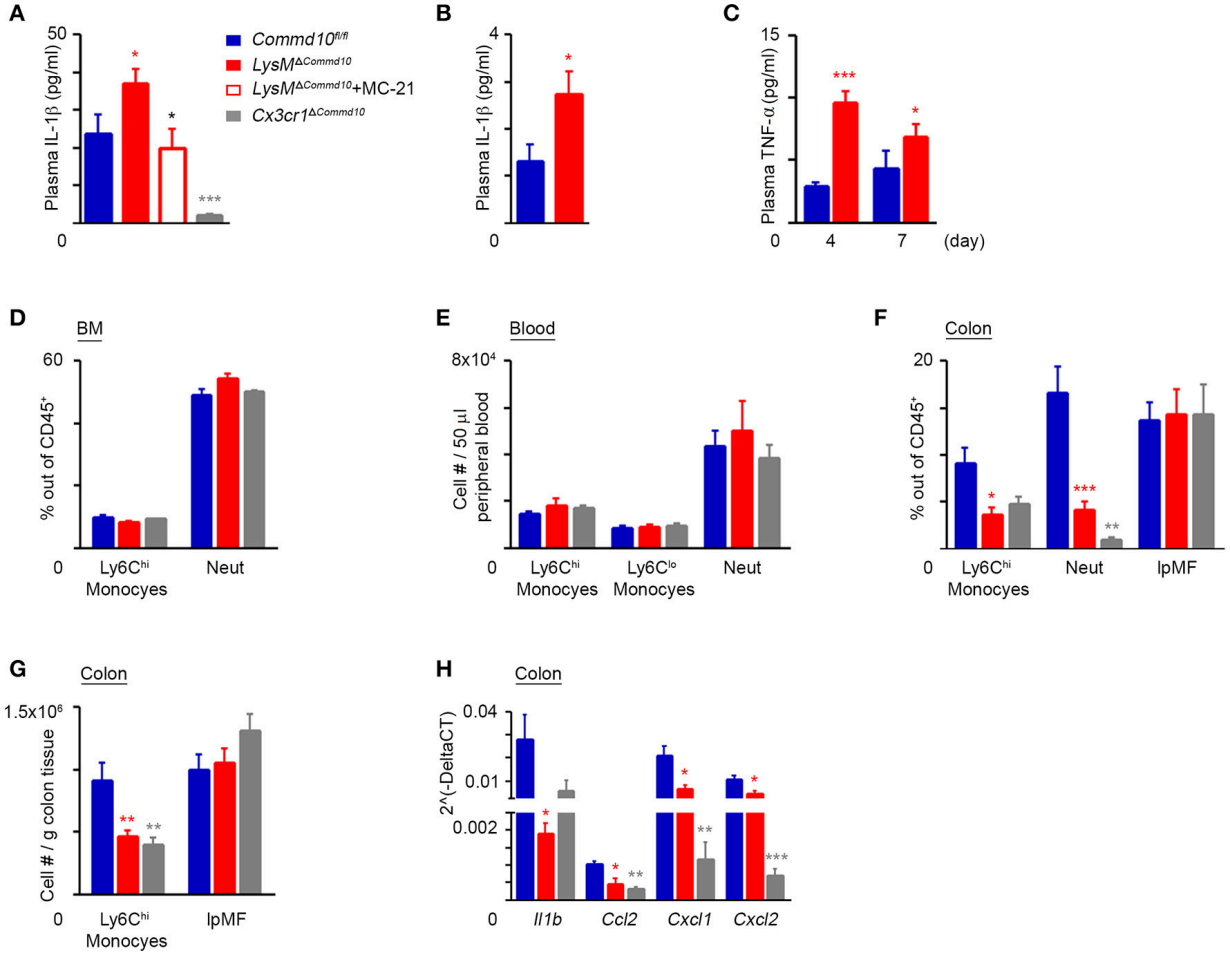
COMMD10 negatively regulates inflammasome activity in Ly6C^hi^ monocytes during colitis. *Commd10*^*fl*/*fl*^ (blue), *LysM*^Δ*Commd*10^ (red) or *Cx3cr1*^Δ*Commd*10^ (gray) mice were treated with DSS (1.5% in drinking water) for 4 or 7 days. Where indicated, MC-21 was injected every day, starting at 24 h after DSS administration (red border). **(A)** ELISA analysis of plasma IL-1β at day 4 and **(B)** day 7 of DSS (*n* ≥ 5). **(C)** ELISA analysis of plasma TNF-α (n≥4). **(D)** Flow cytometry-based assessment of BM Ly6C^hi^ monocyte and neutrophil fractions out of total CD45^+^ immune cells at day 4 of DSS (*n* ≥ 5). **(E)** Flow cytometry-based assessment of Ly6C^hi^ and Ly6C^lo^ monoyctes and neutrophils in 50 μl peripheral blood at day 4 of DSS (*n* = 6). **(F)** Flow cytometry-based assessment of colonic Ly6C^hi^ monocyte, neutrophil and resident lpMF fractions out of total CD45^+^ immune cells at day 4 of DSS (*n* = 6). **(G)** Flow cytometry-based assessment of colonic Ly6C^hi^ monocyte and resident lpMF abundance normalized to tissue mass at day 4 of DSS (*n* ≥ 10). **(H)** qRT-PCR gene expression of *Il1b, Ccl2, Cxcl1*, and *Cxcl2* in colon tissue at day 4 of DSS (*n* ≥ 4). Data were analyzed by unpaired, two-tailed *t-*test, comparing each time between one of the mouse groups with *Commd10*^*fl*/*fl*^ mice (colored stars), or between *LysM*^Δ*Commd*10^ mice with and without MC-21 treatment (black stars). Results are presented as mean ± SEM with significance: ^*^p < 0.05, ^**^p < 0.01, ^***^p < 0.001. Data in **(A,C,F–H)** represent two independent experiments. Data in **(D** and **E)** represent a single experiment.

Colonic infiltrating Ly6C^hi^ monocytes become the dominant pro-inflammatory myeloid effector cells at day 4 of DSS-induced colitis (10). Therefore, we next investigated the effect of COMMD10-deficiency on Ly6C^hi^ monocyte inflammasome activity at this time point. Similarly to their response to LPS-induced systemic inflammation (Figure 3A), *LysM*^Δ*Commd10*^ mice exhibited a prominent increase in plasma levels of IL-1β, which was completely ameliorated upon MC-21-governed Ly6C^hi^ monocyte ablation. Further emphasizing that Ly6C^hi^ monocytes are the main source for the augmented plasma IL-1β production, *Cx3cr1*^Δ*Commd10*^ mice exhibited a significant reduction in plasma IL-1β, even in comparison to control *Commd10*^*fl*/*fl*^ mice. Thus, these results exclude a contribution of tissue-resident macrophages to the overall increase of plasma IL-1β observed in the *LysM*^Δ*Commd10*^ mice (Figure 6A). At day 7, the augmented plasma IL-1β production in *LysM*^Δ*Commd10*^ mice was still apparent (Figure 6B). Plasma TNFα levels were also elevated in the *LysM*^Δ*Commd10*^ mice at day 4 and 7 (Figure 6C). We next examined whether generation of Ly6C^hi^ monocytes and neutrophils in the BM or their egress to the circulation were affected by the specific COMMD10-deficiencies in *LysM*^Δ*Commd10*^ and *Cx3cr1*^Δ*Commd10*^ mice. However, flow cytometry analysis of BM and blood of these transgenic mice at day 4 of DSS-induced colitis revealed no clear differences in their levels (Figures 6D,E). Hence, these results suggest that Ly6C^hi^ monocyte hyper-inflammasome activity, rather than availability in the circulation, is responsible for the increased plasma levels of IL-1β in *LysM*^Δ*Commd10*^ mice. Surprisingly in the colon, there were significantly reduced fractions of Ly6C^hi^ monocytes and neutrophils in both *LysM*^Δ*Commd10*^ and *Cx3cr1*^Δ*Commd10*^ mice, with no clear difference in the abundance of colonic lpMFs (Figure 6F). This was further corroborated by showing reduced number per tissue mass of colonic Ly6C^hi^ monocytes, but not of lpMFs, in both *LysM*^Δ*Commd10*^ and *Cx3cr1*^Δ*Commd10*^ mice (Figure 6G). In agreement with this, the colonic expression of the genes encoding for the Ly6C^hi^ monocyte and neutrophil recruitment chemokines *Ccl2, Cxcl1*, and *Cxcl2*, respectively, was markedly reduced in both mice (Figure 6H). Finally, *LysM*^Δ*Commd10*^ colons exhibited reduced local transcription of *Il1b* gene, which is in alignment with the declined accumulation of Ly6C^hi^ monocytes (Figure 6H). Therefore, these results suggest that circulating Ly6C^hi^ monocytes are responsible for the augmented systemic production of IL-1β.

### COMMD10 is downregulated in mouse Ly6C^hi^ monocytes and their equivalent human CD14^hi^ monocytes during intestinal inflammation

**Figure 7:**
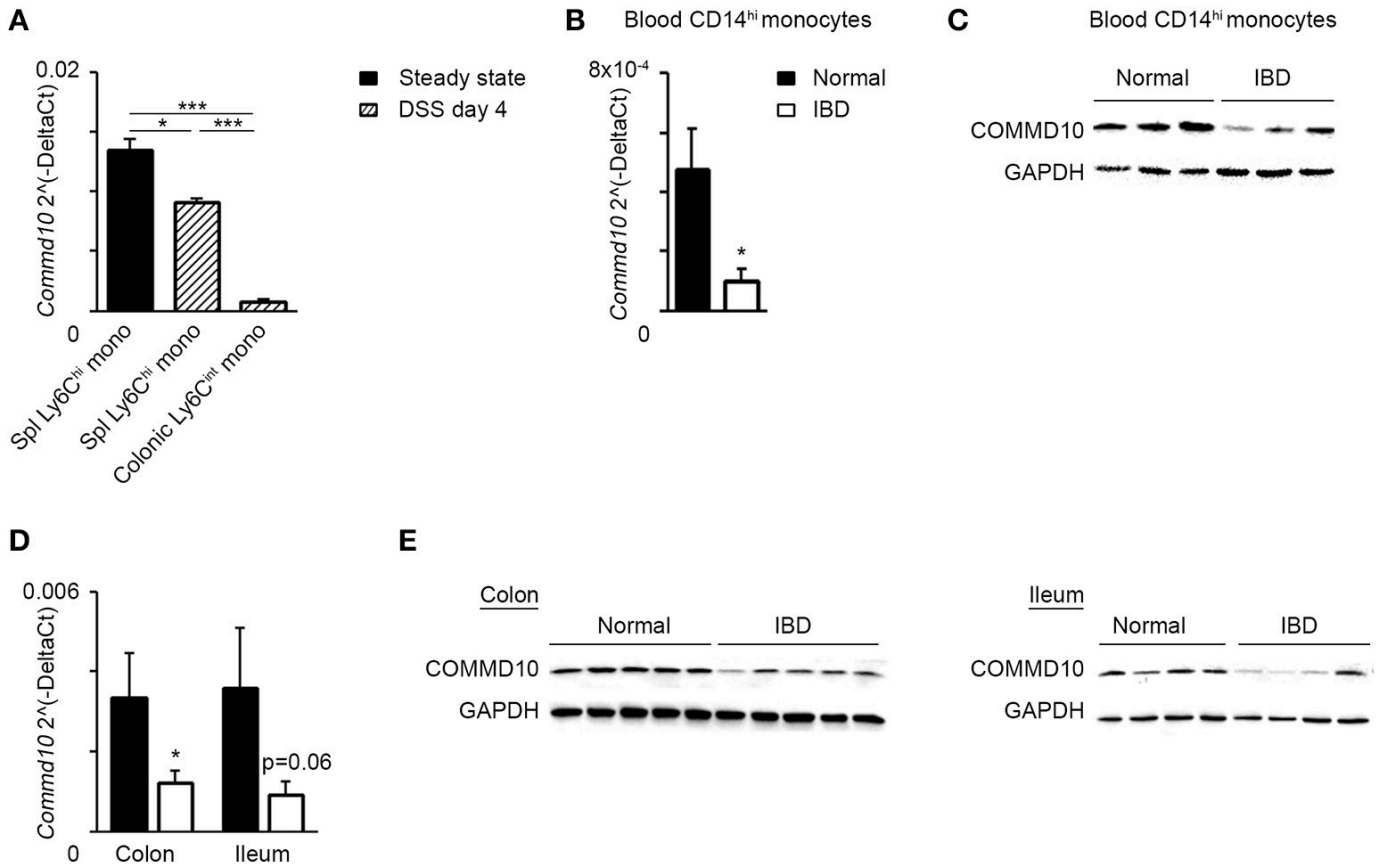
COMMD10 is downregulated in mouse Ly6C^hi^ monocytes and their equivalent human CD14^hi^ monocytes during intestinal inflammation. **(A)** Splenic and colonic Ly6C^hi^ monocytes were sorted from steady state mice (solid) or at day 4 of DSS (diagonal). **(B,C)** Circulating CD14^hi^ monocytes were sorted from patients with active IBD (black border) or healthy controls (solid). **(B)** qRT-PCR gene expression of *Commd10* and **(C)** immunoblot demonstrating COMMD10 protein expression. GAPDH was utilized as control (*n* ≥ 3). **(D,E)** Biopsies were obtained from colons and terminal ileums of IBD patients with active disease and healthy controls. **(D)** qRT-PCR analysis of *Commd10* gene expression. **(E)** Immunoblots demonstrating COMMD10 protein expression in colon (left panel) and ileum (right panel) biopsies obtained from IBD patients vs. healthy controls. GAPDH was utilized as controls (*n* ≥ 14). Data were analyzed by unpaired, two-tailed *t-*test, comparing each time between IBD with healthy controls. Results are presented as mean ± SEM with significance: ^*^p < 0.05, ^***^p < 0.001. Data in **(A–C)** represent a single experiment.

The results above highlight an important negative immunoregulatory role for COMMD10 in tuning Ly6C^hi^ monocyte-driven inflammation during colitis (Figure 6). Therefore, downregulation of COMMD10 levels may be essential to license their inflammatory behavior. Indeed, in comparison to Ly6C^hi^ monocytes sorted from steady state splenic reservoirs, there was a profound decrease in the transcription of *Commd10* in their Ly6C^hi^ monocyte counterparts sorted from splenic reservoirs and inflammatory colons at day 4 of DSS-induced colitis (Figure 7A). Furthermore, *Commd10* gene expression was also profoundly reduced in the transition of Ly6C^hi^ monocytes from circulation (splenic reservoir) to colonic tissue in the context of colitis (Figure 7A). A functionally similar pro-inflammatory CD14^hi^ monocyte population has been identified within inflamed tissues of IBD patients (36, 37). Notably, there was also significant decline in the expression of COMMD10 gene (Figure 7B) and protein (Figure 7C) in circulating CD14^hi^ monocytes sorted from patients with active IBD vs. healthy subjects. This reduction in COMMD10 expression was further evident in colonic or ileal tissue biopsies extracted from IBD patients vs. healthy subjects at both gene (Figure 7D) and protein (Figure 7E) levels.

## Discussion

We have identified a novel role for COMMD10, a protein with unknown function, in the regulation of Ly6C^hi^ monocyte-driven inflammation during systemic inflammation. In particular, lack of COMMD10 in circulating and tissue-infiltrating Ly6C^hi^ monocytes leads to augmented inflammasome-induced caspase-1 and 11 activity during LPS-induced systemic inflammation. The hyper-inflammatory activity of COMMD10-deficient monocytes compromises intestinal barrier function culminating in increased bacterial translocation to internal organs including the liver. In the setting of colitis, we demonstrate that disrupted COMMD10 activity unleashes the inflammatory behavior of circulating Ly6C^hi^ monocytes. Our results also indicate for the first time the involvement of COMMD10 in the inhibition of NF-κB activation in monocytes and their macrophage descendants.

Inflammasomes are key signaling platforms that detect pathogenic microorganisms and sterile stressors, and activate in response the highly pro-inflammatory cytokine IL-1β (14, 15). This cytokine is controlled by two checkpoints: NF-κB-driven transcription of pro-IL-1β as well as inflammasome-dependent caspase-1-mediated maturation and release. An additional non-canonical inflammasome activation pathway has been described in macrophages responding to Gram^neg^ bacteria, by which caspase-11 directly senses cytosolic LPS to induce IL-1β secretion (16). Utilizing an unbiased multiplex cytokine and chemokine array we show that COMMD10-deficiency in myeloid cells leads to a profound elevation in plasma levels of various pro-inflammatory cytokines, including IL-1β, during endotoxemia. This was further consolidated by a specific ELISA analysis for IL-1β in the plasma and activation of caspase-1 and 11 in the liver, spleen and colon. Strikingly, this phenotype was abolished upon the specific ablation of Ly6C^hi^ monocytes. Moreover, *Cx3cr1*^Δ*Commd10*^ mice, in which COMMD10-deficiency is mostly targeted to tissue-resident macrophages (26), showed similar inflammasome activity to that of control *Commd10*^*fl*/*fl*^ mice. COMMD10-deficiency had no effect also on inflammasome activity in isolated BM neutrophils responding to LPS/ATP stimulation. In alignment, we demonstrate that *Commd10* gene expression is significantly higher in circulating Ly6C^hi^ monocytes vs. neutrophils. Therefore, these results point to COMMD10 as a negative regulator of both canonical and non-canonical inflammasome pathways uniquely in Ly6C^hi^ monocytes among the various phagocytic cells targeted by *Lyz2*^*cre*^. Synthesis and release of IL-1β differ between monocytes and macrophages as the former have constitutively activated caspase-1, and sole transcriptional stimulation by bacterial ligands, such as LPS leads to the release of active IL-1β (18). In this respect, we show that LPS stimulation is solely sufficient to induce augmented IL-1β transcription and secretion in both COMMD10-deficient Ly6C^hi^ monocyte-derived BMDM and COMMD10-deficient sorted circulating Ly6C^hi^ monocytes.

Several studies have linked members of the COMMD protein family to transcription factor regulation. The first transcription factor to be associated with COMMD proteins was NF-κB (21, 28), a master regulator of inflammation and cell survival. COMMD1 is a negative regulator of this pathway, acting to promote the ubiquitination and proteasomal degradation of transcriptionally active NF-κB subunits (38). Consistent with this, COMMD1 deficiency in myeloid cells results in increased NF-κB activation, thereby exacerbating the course of LPS-induced systemic inflammation (24). Studies in non-immune cells genetically manipulated to overexpress COMMD10 have indicated its ability to inhibit NF-κB-mediated transcription as well (21), though the physiological relevance of this finding in general, and in immune cells in particular, remains elusive. Here we show that COMMD10-deficiency in Ly6C^hi^ monocytes and their macrophage descendants, as well as in their human CD14^hi^ monocyte equivalents, leads to increased and persistent activation of NF-κB in response to LPS or *E. coli* infection. This may explain the augmented transcription of NF-κB-target genes in these cells. Moreover, the unleashed NF-κB activity in COMMD10-deficient Ly6C^hi^ monocytes and their macrophage progenies may also be responsible for the increased inflammasome activation in these cells. Of particular interest is the increased expression of pro-caspase-11 in the *LysM*^Δ*Commd10*^ livers following LPS treatment, which was abolished by Ly6C^hi^ monocyte ablation. Pro-caspase-11 transcription via LPS-TLR4 signaling can be induced in parallel by either MyD88-NF-κB or Trif-IRF3 signaling (39). While we provide evidence for increased NF-kB involvement, the potential contribution of the Trif-IRF3 pathway requires further studies. COMMD1 suppresses NF-κB activity by promoting the ubiquitination and degradation of its subunits (38). Yet, the mechanism coupling COMMD10 to NF-κB inhibition remains unknown. An additional transcriptional program shown to be influenced by COMMD1 is hypoxia inducible factor (HIF). Accordingly, constitutional deficiency of COMMD1 leads to broad activation of HIF-1α and embryonic lethality (40), while its deficiency in cancer cells promotes HIF-1α-dependent gene expression and tumor cell invasion (41). Sepsis is characterized by a dysregulated inflammatory response to infection (42). Interestingly, monocytes from septic patients acquire an immunosuppressive phenotype by upregulating HIF-1α activity (43). With respect to COMMD10, there is no evidence that it is involved with HIF-1α regulation. Overall, further studies are needed to delineate the task division between distinct COMMD proteins in shaping the transcriptional programing of Ly6C^hi^ monocytes in response to endotoxin stimulation. Importantly also, given the role of different COMMD proteins in mediating intracellular protein trafficking events (22, 25), COMMD10 may potentially regulate the inflammasome complex activity by affecting its assembly or stability.

*LysM*^Δ*Commd10*^ mice exhibit increased mortality in response to systemic LPS challenge. Intestinal barrier function may be disrupted by various pro-inflammatory cytokines, such as IL-1β (31, 32), leading to systemic bacterial dissemination. Therefore, the augmented pro-inflammatory cytokine response in the *LysM*^Δ*Commd10*^ mice at 4 h after LPS challenge, and in particular the systemic elevated inflammasome activation and IL-1β production by Ly6C^hi^ monocytes, may compromise intestinal epithelial cell layer integrity. Indeed, we show severe intestinal barrier leakage in the *LysM*^Δ*Commd10*^ mice and reduced transcription of TJ genes in the colon. The ablation of Ly6C^hi^ monocytes partially restores intestinal barrier function and the expression of selected TJ genes, further confirming a role for COMMD10-deficient monocytes in impairing gut barrier function. IFNγ and TNFα are additional pro-inflammatory cytokines known to increase intestinal TJ permeability (44). The expression of both was also significantly higher in the plasma of LPS-challenged *LysM*^Δ*Commd10*^ mice and supernatants of *LysM*^Δ*Commd10*^ BMDM, therefore suggesting their possible contribution to the observed intestinal barrier disruption in these mice. An alternative explanation for the increased bacterial dissemination and mortality in LPS-challenged *LysM*^Δ*Commd10*^ mice may be that over-activation of caspases-1 and –11 in these mice facilitates pyroptosis. This lytic form of cell death releases DAMPs that can further incite multiple organ dysfunction and mortality, and also lead to deficiency in intestinal epithelial cells and various immunocytes (45).

We and others have indicated a pivotal role for Ly6C^hi^ monocytes and their CD14^hi^ human counterparts in the initiation and propagation of intestinal inflammation (10, 13, 36, 37). Our results mark COMMD10 as a negative regulator of inflammasome activity in these cells in the context of colitis as well. We also demonstrate that COMMD10 expression is reduced in circulating Ly6C^hi^ monocytes and their equivalent human CD14^hi^ monocyte subset in the context of gut inflammation. Hence, regulation of COMMD10 expression or activity may be an essential mechanism to tune the physiological inflammasome activity in these cells. The increased colitis score observed in the *LysM*^Δ*Commd10*^, but not in *Cx3cr1*^Δ*Commd10*^ mice, further substantiates that COMMD10-governed immunoregulation is important already at the level of these circulating monocytes. Surprising was the finding of reduced numbers of effector Ly6C^hi^ monocytes and neutrophils in the inflamed colons in both *LysM*^Δ*Commd10*^ and *Cx3cr1*^Δ*Commd10*^ mice vs. the control *Commd10*^*fl*/*fl*^ mice. A possible explanation may be that COMMD10-deficiency disturbs the migratory capacity of Ly6C^hi^ monocytes and neutrophils. However, our data showing similar representation of Ly6C^hi^ monocytes in the BM and blood compartments of these mice argue against any affect for COMMD10-deficiency on their generation or migration to the circulation. The low expression of *Commd10* gene in neutrophils decreases the probability of an intrinsic effect for COMMD10-deficiency on the migration of these cells. Furthermore, the paucity of Ly6C^hi^ monocytes and neutrophils in the *Cx3cr1*^Δ*Commd*10^ colons also excludes cell-intrinsic migratory effects of COMMD10-deficiency, as in these mice cre-mediated recombination (and ensuing COMMD10-deficiency) is mainly targeted to resident macrophages (26). Instead, their paucity in both *LysM*^Δ*Commd10*^ and *Cx3cr1*^Δ*Commd10*^ mice is in alignment with reduced colonic expression of their recruiting chemokines in these mice. We have previously shown that colonic lpMFs express these chemokines at day 4 of DSS (10). Therefore, COMMD10-deficiency may have an anti-inflammatory effect in colonic-resident lpMFs. This argument is also supported by the profound reduction in plasma IL-1β in the *Cx3cr1*^Δ*Commd*10^ vs. *Commd10*^*fl*/*fl*^ mice. Further studies are required to delineate the immunoregulatory circuits governed by COMMD10 in resident lpMF.

Robust, and yet balanced inflammatory responses are involved in detection of tissue injury or infection, subsequent inflammatory reaction, and ultimate resolution. Monocytes seem perfectly suited for this task since they are equipped with a large repertoire of scavenger and pattern recognition receptors enabling them to rapidly react to local danger signals (46). Their high plasticity empowers their prompt molecular adaptation in response to altering cues and production of effector molecules capable of driving both inflammation and resolution (1, 3). Although the recent past has seen great advancement in our comprehension of Ly6C^hi^ monocyte biology, the molecular mechanisms that allow monocytes to act in restorative rather than pathological manner remain largely elusive. Here we report on the important role of COMMD10 in restraining Ly6C^hi^ monocyte-driven inflammation.

## Author contributions

The corresponding authors (CV and NG) confirm that all authors agree to be accountable for the content of the work. OM, SB, NG, and CV designed, performed, and analyzed all experiments and wrote the manuscript. KC, IF, and SG substantially assisted OM and SB with some of the major *in vivo* experiments and gene expression analyses. NM and ZH greatly assisted with the recruitment and collection of IBD patient samples. EB contributed to the design of the original idea in this manuscript.

### Conflict of interest statement

The authors declare that the research was conducted in the absence of any commercial or financial relationships that could be construed as a potential conflict of interest.
