# Supplementary material for: Impaired COMMD10-Mediated Regulation of Ly6Chi Monocyte-Driven Inflammation Disrupts Gut Barrier Function
Source: Front Immunol. 2018 Nov 14;9:2623. doi: 10.3389/fimmu.2018.02623 (PMC6246736; doi:10.3389/fimmu.2018.02623)
Supplement: Supplementary file 2 [file Data_Sheet_1.docx]

Supplementary Material

**Impaired COMMD10-Mediated Regulation of Ly6C^hi^ Monocyte-Driven Inflammation Disrupts Gut Barrier Function**

Odelia Mouhadeb^1,2†^, Shani Ben Shlomo^1†^, Keren Cohen^1,2^, Inbal Farkash^1^, Shlomo Gruber^1^, Nitsan Maharshak^1^, Zamir Halpern^1^, Ezra Burstein^3,4^, Nathan Gluck1^*‡^ and Chen Varol ^1,2*‡^

^1^ The Research Center for Digestive Tract and Liver Diseases, Tel-Aviv Sourasky Medical Center and Sackler School of Medicine, Tel-Aviv University, Israel.

^2^ Department of Clinical Microbiology and Immunology, Sackler School of Medicine, Tel-Aviv University, Israel.

^3^ Department of Internal Medicine and Department of Molecular Biology, University of Texas Southwestern Medical Center, Dallas, USA

^†^ These authors contributed equally

^‡^ These authors share senior authorship

^*^ Co-senior author

**Correspondence:**

Dr. Chen Varol: [chenv@tlvmc.gov.il](mailto:chenv@tlvmc.gov.il)

Dr. Nathan Gluck: [nathang@tlvmc.gov.il](mailto:nathang@tlvmc.gov.il)

# Supplementary Figures and Tables

**Figure S1**

**
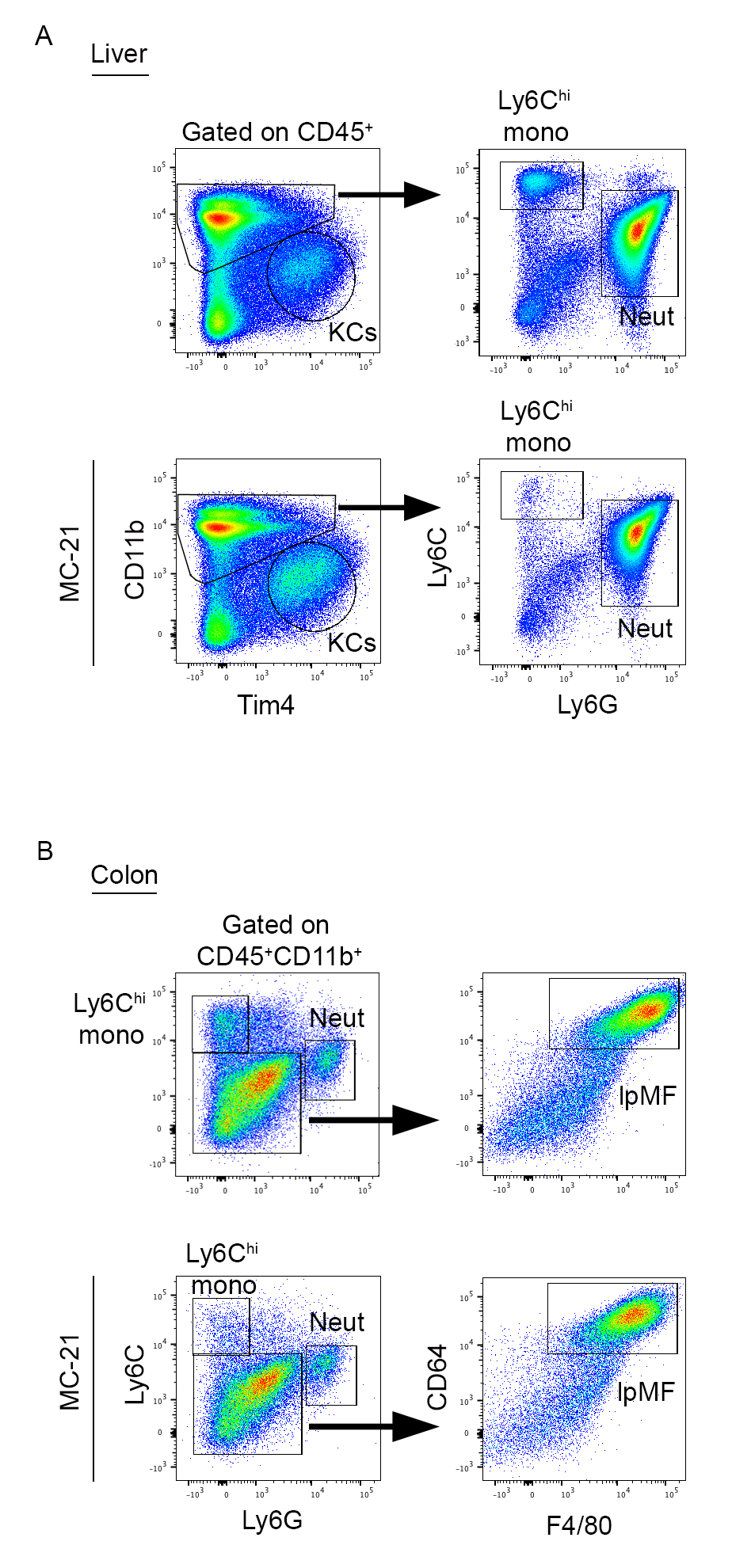
**

**Figure S1. Inducible depletion of infiltrating Ly6C^hi^ monocytes using the anti-CCR2 MC-21 antibody**

*LysM^ΔCommd10^* mice were i.p. injected with LPS (0.2 mg per mouse of similar weight). Some of the mice were treated with MC-21 12 h prior to LPS stimulation. (**A**) Representative flow cytometry images showing gating strategy of liver Ly6C^hi^ monocytes, KCs and neutrophils out of CD45^+^ non-parenchymal immune cells**.** Top panels: *LysM^ΔCommd10^* mice treated with LPS alone. Bottom panels: *LysM^ΔCommd10^* mice treated with LPS and MC-21. (**B**) Representative flow cytometry images showing gating strategy of colonic Ly6C^hi^ monocytes, resident lpMFs and neutrophils out of CD45^+^CD11b^+^ immune cells. Top panels: *LysM^ΔCommd10^* mice treated with LPS alone. Bottom panels: *LysM^ΔCommd10^* mice treated with LPS and MC-21.

**Figure S2.**


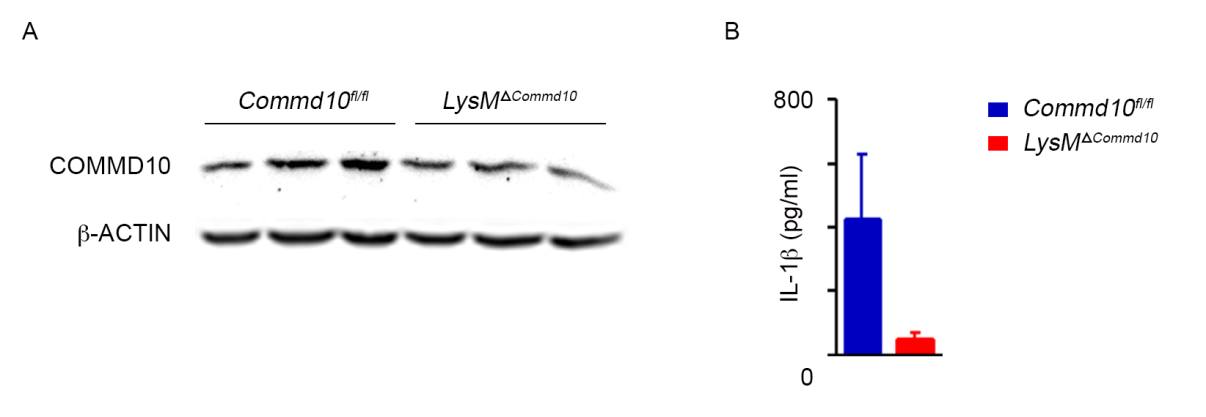


**Figure S2. LPS-challenged COMMD10-deficient BM neutrophils exhibit declined production of IL-1β.**

Neutrophils were isolated from BM of *Commd10^fl/fl^* (blue) or *LysM^ΔCommd10^* (red) mice and subjected to LPS (100ng/ml) for 3h. ATP was added in the last 30 min of the experiment. (**A**) Immunoblots showing the expression of COMMD10. β-actin was utilized as control (n=3). (**B**) ELISA analysis of IL-1β from cell free supernatants (n=3). Data were analyzed by unpaired, two-tailed *t-test*, comparing *Commd10^fl/fl^* and *LysM^ΔCommd10^* and are presented as mean ± SEM. Data in panels A and B represent a single experiment.

**Figure S3.**

**
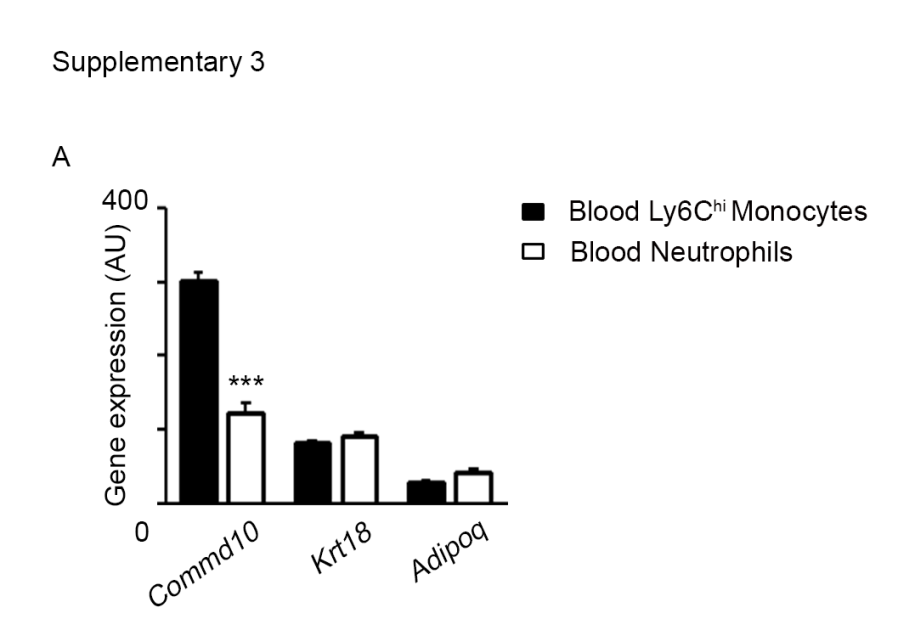
**

**Figure S3. *Commd10* gene expression is near background levels in blood neutrophils and significantly higher in blood Ly6C^hi^ monocytes**

Bar graph showing the raw gene expression of COMMD10 (*Commd10*) in comparison with that of the adipocyte marker adiponectin (*Adipoq*) and the hepatocyte and cholangiocyte marker Cytokeratin18 (*Krt18*), both were used to set background expression levels. Gene-expression data were extracted from the ImmGen Consortium database (GSE37448) (n=3 for Ly6C^hi^ monocytes, n=4 for blood neutrophils). Data were analyzed by unpaired, two-tailed *t-test*, comparing blood Ly6C^hi^ monocytes and blood neutrophils and presented as mean ± SEM with significance: *** p < 0.001.

**Table S1.**


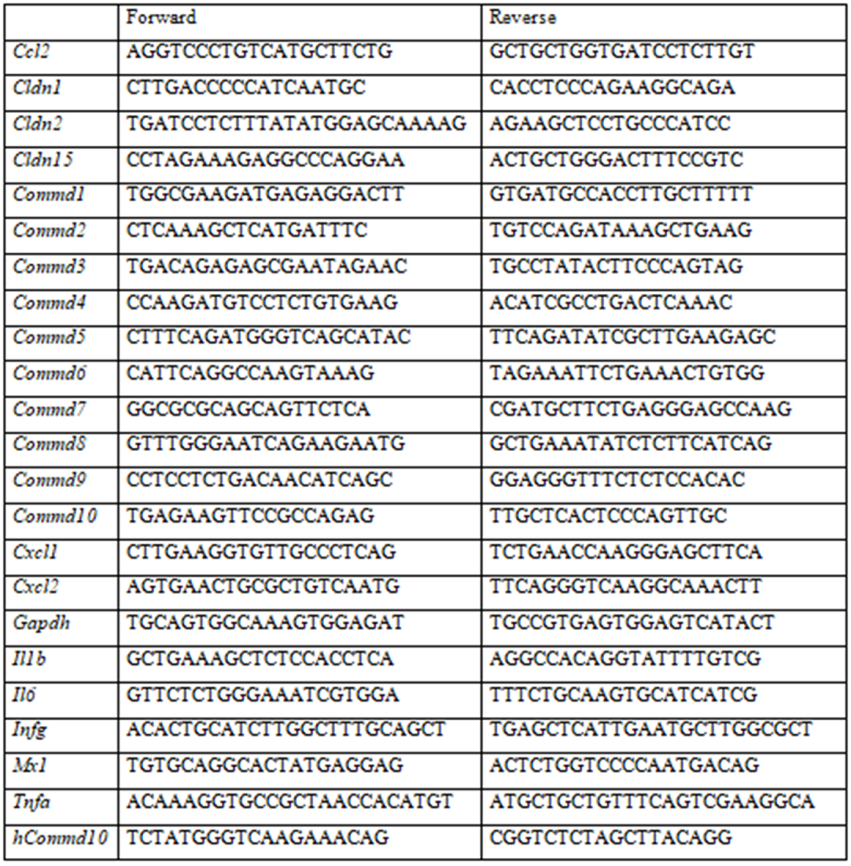


**Table S1. List of primers**

A table showing the sequences of all primers used.

**Movie S1. *LysM*^Δ^*^Commd10^* but not *Cx3cr1*^Δ^*^Commd10^* mice exhibit increased intestinal inflammation during DSS-induced colitis**

*Commd10^fl/fl^*, *LysM*^Δ^*^Commd10^* or *Cx3cr1*^Δ^*^Commd10^* mice were treated with DSS (1.5% in drinking water) for 7 days. Colonoscopy movie showing representative captures of the five parameters which indicate colitis severity grade: thickening of the colon wall, changes in the normal vascular pattern, presence of fibrin, mucosal granularity and stool consistency**.**
